# Supplementary figures and images for: Downregulation of both mismatch repair and non-homologous end-joining pathways in hypoxic brain tumour cell lines
Source: PeerJ. 2021 Apr 30;9:e11275. doi: 10.7717/peerj.11275 (PMC8092103; doi:10.7717/peerj.11275)

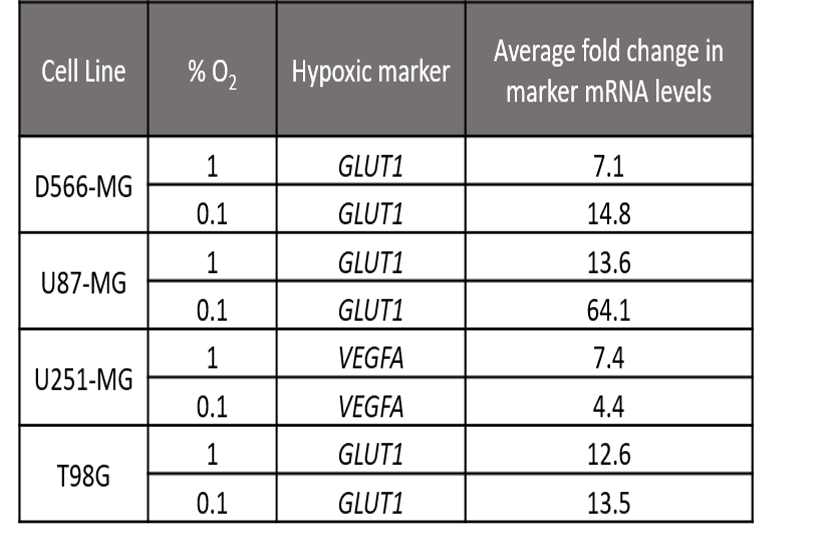

Supplement: Supplemental Information 1 — For all samples submitted for gene expression analysis by NanoString, the expression of GLUT1 was measured by RT-PCR. mRNA levels of GLUT1 are displayed as a fold change with respect to levels in 21% O2. Data are an average of three independent experiments [file peerj-09-11275-s001.png]

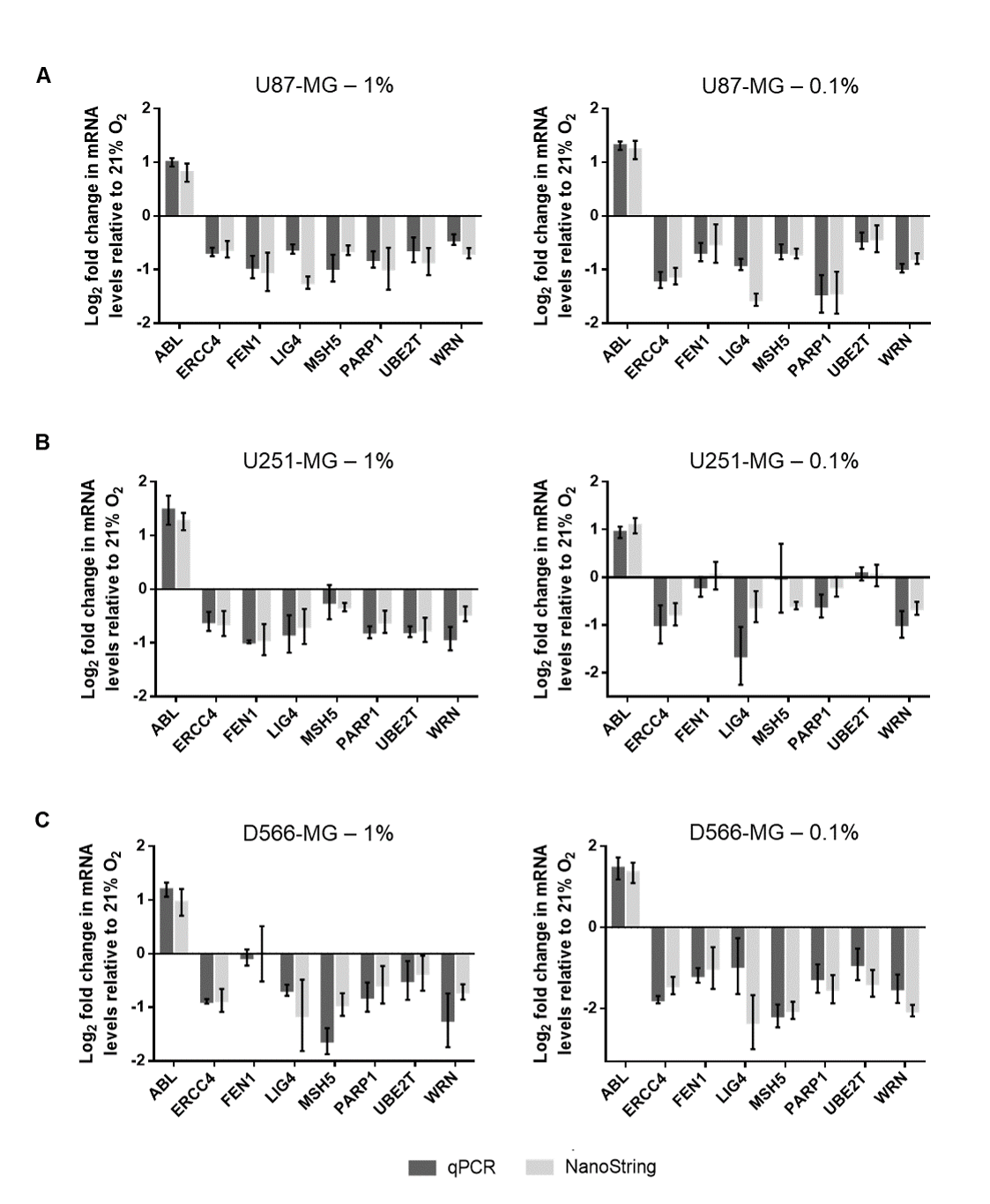

Supplement: Supplemental Information 2 — Seven genes were selected from the NanoString panel for validation by RT-PCR. cDNA samples for (A)U87-MG, (B) U251-MG and (C) D566-MG was utilised in RT-PCR experiments to assess the expression levels of selected genes. Data are expressed as log2 fold change with respect to expression at 21% O2. Data from NanoString is included for direct comparison. RT-PCR data is represented as the mean of three independent experiments. [file peerj-09-11275-s002.png]

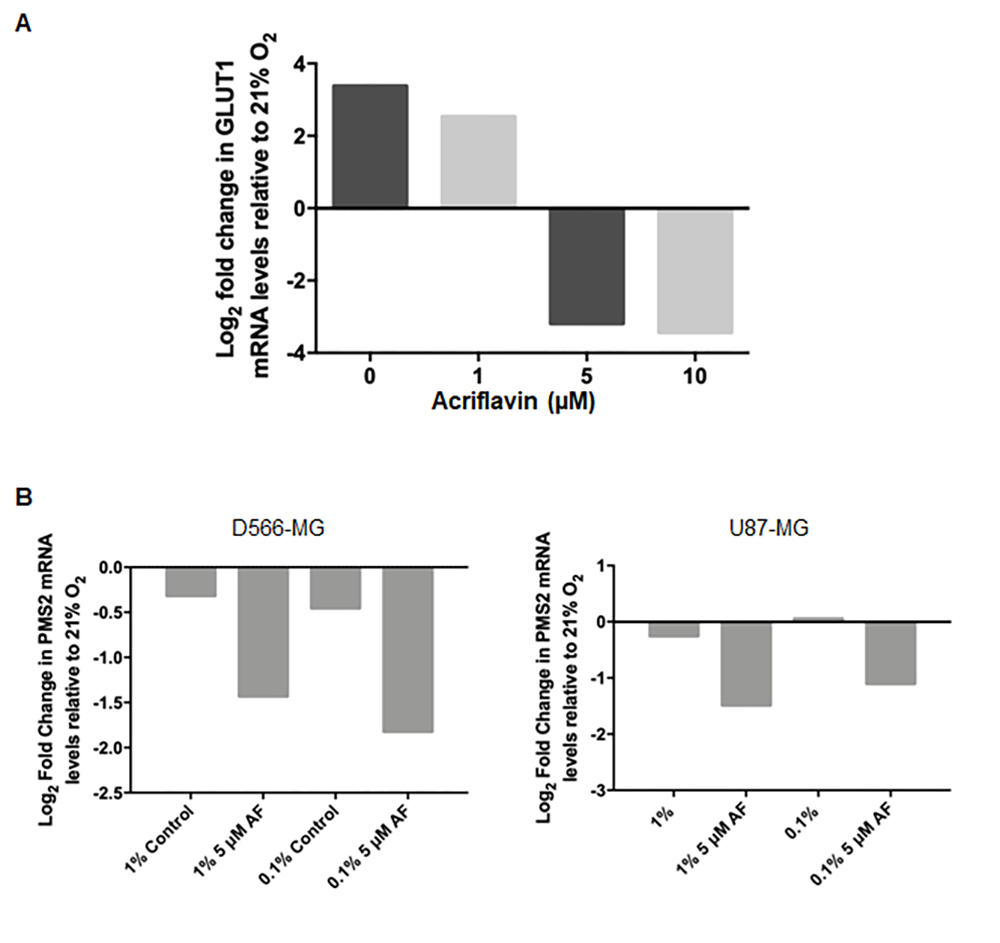

Supplement: Supplemental Information 3 — (A) D566-MG cells were incubated in 0.1% O2 and treated with 0–10 µM Acriflavin for 24 h. Control cells remained in 21% O2. RT-PCR was performed to assess GLUT1 expression levels. Data represent the fold change in GLUT1 expression relative to 21% O2. Data are from a single experiment. (B) HIF inhibition does not restore PMS2 mRNA levels in hypoxia. D566‑MG and U87-MG cells were incubated in 21% and 1% O2 for 24 h with and without 5 µM Acriflavin, the plots show the log2 fold change in mRNA levels with respect to levels for the 21% O2 samples. Data are the mean of at least two independent experiment. [file peerj-09-11275-s003.png]

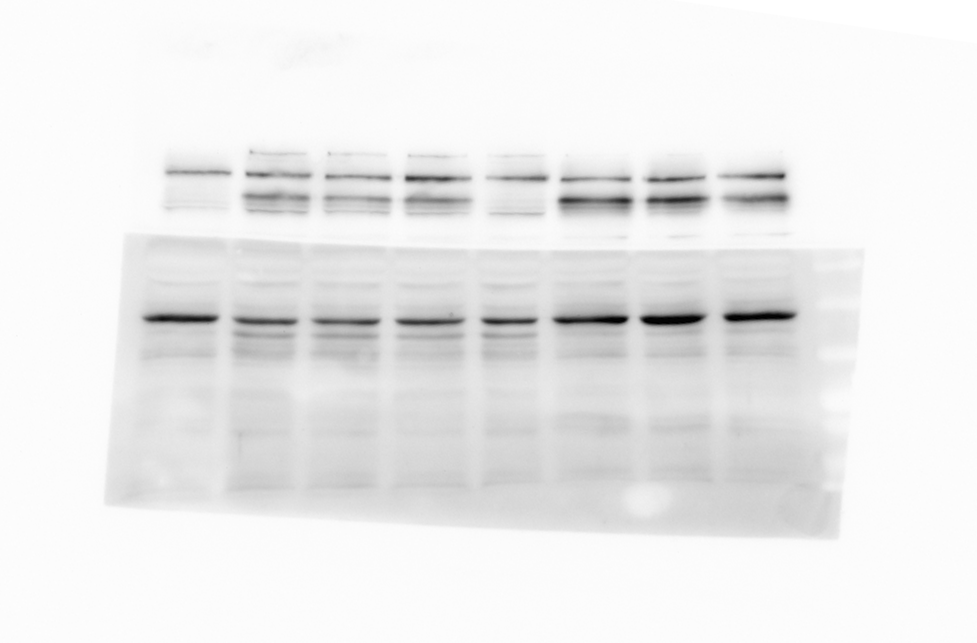

Supplement: Supplemental Information 4 [file peerj-09-11275-s004.zip › Uncropped blots/Fig 5 HIF1 and Actin blots.png]

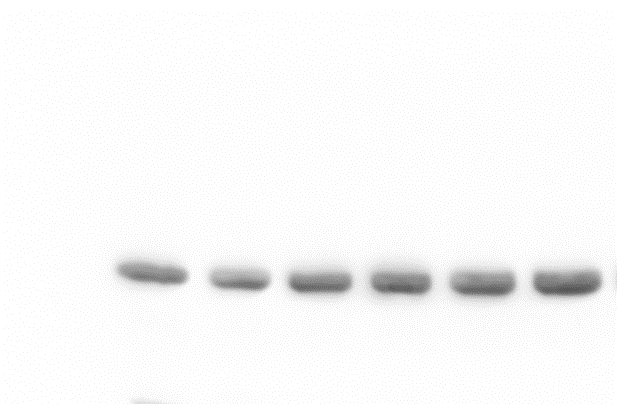

Supplement: Supplemental Information 4 [file peerj-09-11275-s004.zip › Uncropped blots/Figure 4 Actin.png]

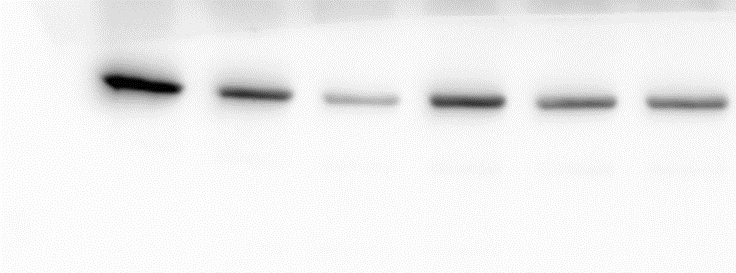

Supplement: Supplemental Information 4 [file peerj-09-11275-s004.zip › Uncropped blots/Figure 4 PMS2.png]

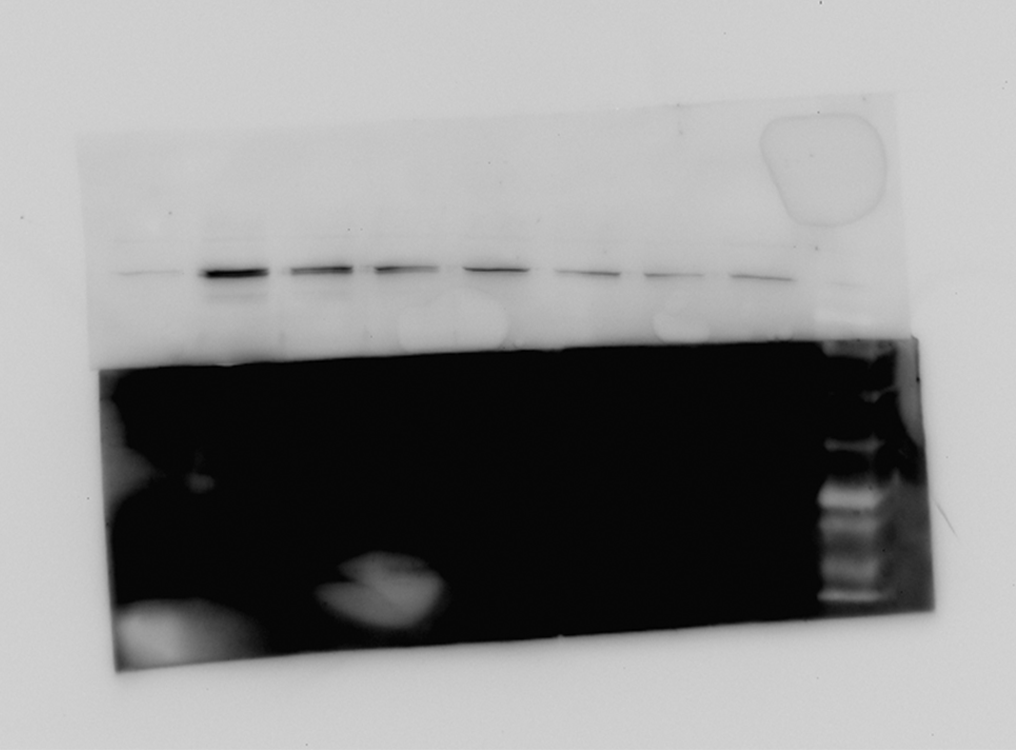

Supplement: Supplemental Information 4 [file peerj-09-11275-s004.zip › Uncropped blots/Figure 5 HIF2 blot.png]
